# Promoting Oral Health Behavior During Pregnancy: A Randomized Controlled Trial

**DOI:** 10.34172/jrhs.2023.119

**Published:** 2023-06-29

**Authors:** Saeid Bashirian, Maryam Barati, Majid Barati, Samane Shirahmadi, Salman Khazaei, Ensiyeh Jenabi, Leila Gholami

**Affiliations:** ^1^Social Determinants of Health Research Center, Hamadan University of Medical Sciences, Hamadan, Iran; ^2^Department of Public Health, School of Public Health, Hamadan University of Medical Sciences, Hamadan, Iran; ^3^Department of Community Oral Health, School of Dentistry, Hamadan University of Medical Sciences, Hamadan, Iran; ^4^Department of Epidemiology, School of Public Health, Hamadan University of Medical Sciences, Hamadan, Iran; ^5^Autism Spectrum Disorders Research Center, Hamadan University of Medical Sciences, Hamadan, Iran; ^6^Department of Periodontology, School of Dentistry, Hamedan Medical Science University, Hamedan, Iran

**Keywords:** Pregnancy, Oral health, Randomized controlled trial, Health promotion

## Abstract

**Background:** Pregnant women are vulnerable to oral disease due to physiological, hormonal, and dietary alterations. The aim of the present study was to evaluate the impact of the educational program according to the Health Promotion Model (HPM) on the oral health prevention behavior of pregnant women.

**Study Design:** A randomized controlled trial.

**Methods:** This study was performed on 105 pregnant women visiting health centers located in Arak from February to November 2022. The subjects were randomly assigned to intervention (n=54) and control (n=51) groups. A reliable and valid questionnaire according to HPM constructs was used to collect the data. The pre-test was conducted in the groups. The intervention group received the educational program in 9 educational sessions (from 12 to 24 weeks of pregnancy). Then, the post-test was conducted in the 36th week of pregnancy in the groups. Finally, the data were analyzed by SPSS software (version 18) and using independent t-test, paired t-test, and Chi-square test.

**Results:** There were statistically significant differences between the intervention and control groups regarding perceived benefits (24.68±3.63 vs. 26.57±3.67, *P*=0.009), perceived barriers (7.31±3.14 vs. 5.81±3.59, *P*=0.025), positive affect (10.50±1.66 vs. 11.29±1.34, *P*=0.009), negative affect (1.59±0.223 vs. 1.40±1.51, *P*=0.006), commitment to the action plan (4.05±1.92 vs. 4.77±1.50, *P*=0.034), and tooth brushing time (2.29±0.72 vs. 2.74±0.48, *P*<0.001). However, no significant difference was observed regarding the tooth brushing frequency (2.05±0.58 vs. 2.07±0.66, *P*=0.901) after the intervention. The brushing time for 2-3 minutes in the intervention group increased from 51.85% to 75.92% after the intervention.

**Conclusion:** HPM-based education was effective in promoting the duration of tooth brushing in pregnant women. However, it had no effect on the tooth brushing frequency.

## Background

 One of the most important aspects of public health is oral health, which becomes more important during pregnancy. The importance of oral health throughout pregnancy stems from the temporary and long-term impacts on the health of women and their offspring.^1^ Expectant mothers are more vulnerable to oral disease due to physiological, hormonal, and dietary alterations throughout pregnancy.^2^ Periodontal diseases are the most common oral disease during pregnancy (35-100%). Studies have shown that periodontal diseases affect systemic conditions and are regarded as a risk factor for adverse pregnancy outcomes such as preterm birth and low birth weight.^3-5^ Brushing teeth as a healthy habit can aid in the prevention of dental decay and periodontal diseases.^6^ However, the efficiency of tooth brushing relies on a variety of factors such as the brushing frequency, the duration of brushing, and the tooth brushing techniques among others.^7^ Some studies have demonstrated different rates of tooth brushing frequency among pregnant women in various countries.^8-12^ This rate is undesirable in Iran and indicates the need for oral health interventions during pregnancy.^13-16^ A systematic study reported that oral health intervention studies throughout pregnancy are limited.^17^ In Iran, limited interventional studies have been conducted in this regard.^18-24^ On the other hand, social networks have the potential to involve people in health interventions due to various aspects such as social support, empowerment, peer pressure, and interactive exchange of information and feelings.^25^ Nonetheless, social networks have been used in limited oral health interventions during pregnancy.^17^

 Furthermore, an effective educational intervention depends on the appropriate application of behavioral science theories, and in this regard, health behavior modifications should be based on the determinants of oral health behaviors.^26^ The determinants of oral health behavior during pregnancy include the interpersonal level (including social capital, family support, service providers, and similar factors) in addition to the individual level (including inadequate knowledge, low health literacy, and misconceptions among others) and socio-economic factors. Thus, Pender’s Health Promotion Model (HPM) which includes both individual and interpersonal levels was selected as the conceptual framework.^27-31^ It should be noted that most of the oral health intervention studies during pregnancy are focused on the individual level.^17^

 Therefore, due to the unsuitable condition of oral health in pregnant women, the design of the intervention at the individual and interpersonal levels, the implementation of the educational program over an almost long period, and the significant role of social networks in education, the present study was performed to investigate the promotion of oral health behavior during pregnancy based on HPM.

## Methods

 This study was a randomized controlled trial conducted on expectant mothers visiting the health centers of Arak from February to November 2022. It received approval from the Ethics Committee of Hamedan University of Medical Sciences (IR.UMSHA.REC.1399.863). In addition, this study has been approved by the Iranian Registry of Clinical Trials (identifier: IRCT20221228056955N1; https://www.irct.ir). Informed consent was obtained from all the study participants. The sample size was determined to be 104 subjects (52 individuals in each arm of the study) according to the study by Shamsi et al^23^ and considering the obtained impact size (d) of 0.4, the power of the test of 80%, confidence interval of 95% (1-α = 0.95), and the possible dropout of 10%. The inclusion criteria were having a pregnancy record in one of the health centers of Arak, gestational age below 12 weeks, age of 16-46 years, and the ability to use social media. On the other hand, the exclusion criteria were abortion, premature birth, complex problems during pregnancy, poor oral health, and reluctance to continue participating in the study.

 For sampling, health centers were divided according to the five districts of the municipality. Based on the size and population of each district, a few centers in each district were chosen at random (39 centers out of 50). The selected centers were randomly allocated to control (20 centers) and intervention (19 centers) groups. Eventually, the samples from each center were extracted from the Sib system via simple random sampling.

**Figure 1:**
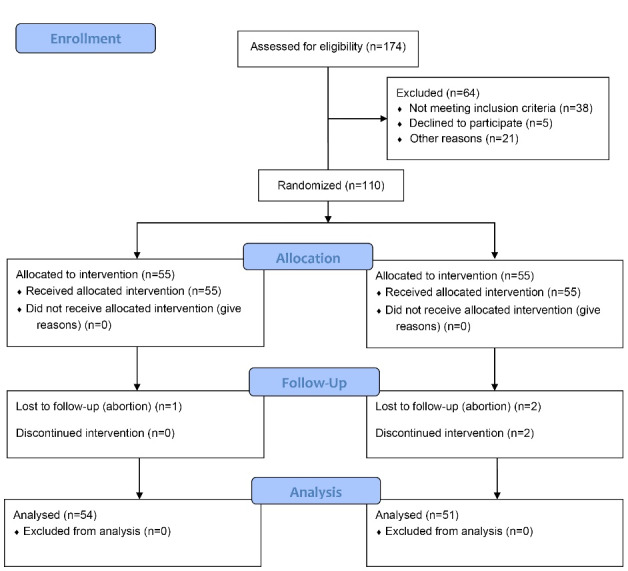
Flowchart of Participants and Group Allocations

 Initially, 174 people were assessed to participate in the study. Nonetheless, 38 people did not meet the inclusion criteria, and 5 people were not interested in participating in the study. Moreover, 21 people did not complete the questionnaire at baseline. Accordingly, 110 people entered the study and were randomly allocated to two intervention and control groups )55 people in each group). During the study, four people in the control group (abortion = 2 and leave the group = 2) and one person in the intervention group (abortion = 1) were excluded from the study. Therefore, 54 people in the intervention group and 51 people in the control group remained for analysis (Figure 1).

 In the present study, the data collection tool was the questionnaire used in the study by Bashirian et al, whose validity and reliability have been measured previously.^31^ The questionnaire consisted of two sections, including demographic data (age, education, number of children, insurance status, and occupation) and questions about HPM constructs. Questions related to model structures included 11 sections.

 The needs assessment and development of the educational program regarding the determinants of tooth brushing behavior in pregnant women were conducted based on the findings of a previous descriptive study.^31^ The educational media included illustrated booklets in pdf format and videos. The scenarios for the videos and the booklet’s contents were developed according to the HPM constructs and by consulting health education and health promotion experts, periodontics specialists, and maternal and child health specialists. Pender’s revised model includes two general components of individuals’ characteristics and experiences, as well as behavior-specific cognitions and affects. Behavior-specific cognitions and affects are perceived benefits, perceived barriers, perceived self-efficacy, affect cues to behavior, interpersonal influencing variables, situational influencing variables, commitment to the action plan, and immediate conflicting demands of behavior.^32^ The prepared media were given to 10 pregnant women, who were requested to share their opinions about them. Using their input, the issues were detected and resolved, making the training material simple and easy to understand. Videos were developed in collaboration with the Media Lab at the Faculty of Health, Hamedan University of Medical Sciences, as well as Hamedan Province Radio and Television.

 Before the beginning of the educational intervention, the pre-test was completed by both groups. Then, the educational program based on HPM was implemented for the intervention group through the social media application WhatsApp in the form of 9 sessions. The objectives of each session were to influence one of the constructs of the HPM. Table 1 presents the strategies employed in the educational intervention based on different constructs of the model. In the intervals between sessions, educational messages were sent regularly and daily to the intervention group in line with educational goals and using illustrated booklets. In addition, training about self-care during pregnancy (topics other than oral health and associated conditions) was provided to the participants in the control group. Actually, the participants were included in the study from the 12th week of pregnancy. The intervention was performed from 12-24 weeks of pregnancy and ended at 24 weeks of pregnancy. The follow-up period was three months, and the participants received no interventions (From 24-36 weeks of pregnancy). Then, in the 36th week of pregnancy, the post-test was conducted in both groups. The telephone numbers of the participants in this study were obtained through comprehensive health service centers. Due to the outbreak of the coronavirus disease 19, the questionnaire was sent to the participants through the WhatsApp application, and they completed the questionnaire. If there was a problem, it was solved by phone call.

**Table 1:** Educational programs to improve the frequency and duration of brushing in pregnant women

**Model construct**	**Methods**	**Strategies**	**Media**
Perceived benefits	Discussion Question and answer	Providing information regarding the association between oral health and systemic condition, the role of tooth brushing in the control and prevention of gum diseases and tooth decay, and the great importance of these conditions during pregnancy as a result of hormonal, dietary, and physiological alterations Group discussion and presentation of evidence and statistics about the advantages of the behavior	Providing booklets and videos through the WhatsApp application
Perceived barriers	Brainstorming Group discussion	Discussion to impact false beliefs (e.g., brushing two times a day causes damage to the gums). Brainstorming about the barriers to brushing your teeth two times a day and at the correct time during pregnancy Brainstorming about ways to overcome these barriers by group admin and the members (including cleaning teeth with a clean cloth, gargling salt water in the mouth twice a day, not brushing with a full stomach, not opening the mouth too much when brushing teeth in order to prevent gag reflex stimulation, and the like) Group discussion about the practicality of these methods	Providing booklets and videos through the WhatsApp application
Perceived self-efficacy	Modeling Verbal persuasion Demonstrating the behavior in smaller steps	Women who brushed their teeth every day despite various hurdles throughout pregnancy shared their experiences in the group During the intervention, verbal persuasion was utilized to convey successful experiences in overcoming difficulties Proper brushing using the modified bass technique was explained step by step in the educational video	WhatsApp social media application
Positive and negative affect cues to behavior:	Expressing the feelings Enhancing the positive emotions	Expressing the feelings caused by tooth brushing. Enhancing the conveyed positive emotions, including the sensation of freshness a consequence of brushing Explaining the cause of negative emotions associated with behavior, including feeling bored	WhatsApp social media application
Interpersonal influencing variables (Social norms and role models)	Networking Improving network linkages Modeling	Creating a WhatsApp group with the participant’s family members, including husbands, mothers, and children, as well as forming a WhatsApp group with the midwives at health centers Improving network linkages via messages from the family of the pregnant woman offering emotional and instrumental support, as well as messages from the midwives at the centers to the pregnant woman offering informational support Providing education about the role of each family member as a role model	WhatsApp social media application
Situational influencing variables	Improving circumstances by leaving clues in the environment	Moving the toothbrush and toothpaste from the bathroom cabinet to the side of the kitchen sink as a reminder for brushing teeth twice a day Providing instruction on how to change the surroundings to prevent the unpleasant conditions linked with brushing throughout pregnancy (e.g., brushing outside the bathroom, sitting on a chair while brushing in the third trimester of pregnancy, and brushing without toothpaste if the pregnant woman is allergic to the smell of toothpaste or suffers from nausea in the first trimester of pregnancy)	WhatsApp social media application
Commitment to the action plan	Goal setting Verbal persuasion	Establishing objectives for brushing teeth two times a day or increasing the brushing time for people who brush their teeth less than two times a day or their brushing time was less than 2-3 minutes a day.	WhatsApp social media application
Immediate competing demands or preferences	Counter conditioning	Addressing actions that instinctively compete with the activity and make it difficult to perform the behavior regularly, and proposing solutions to overcome these barriers (e.g., brushing at any time throughout the day when there is a better feeling and not necessarily at the end of the night and before sleep and at the peak of exhaustion and sleepiness)	WhatsApp social media application
Health-promoting behavior	Skill training	Fully demonstrating brushing with the modified bass technique using the training video and illustrated booklet	WhatsApp social media application

 The outcomes of the study included the constructs of Pender’s HPM (Primary outcomes), as well as the brushing frequency and duration of tooth brushing (Secondary outcomes). Owing to the nature of the study, we could not blind the instructor with regard to the groups, but blinding was considered for the participants, and therefore, this research was a single-blind study.

 The obtained data were analyzed using SPSS software (version 18) via descriptive and inferential statistics (independent *t* test, paired *t* test, and chi-square), and the significance level was considered *P* < 0.05.

## Results

 The mean age in the intervention and control groups was 28.16 ± 5.64 and 28.90 ± 5.52 years, respectively, and the difference was not statistically significant (*P* = 0.666). No statistically significant difference was detected between the intervention and control groups regarding the other demographic factors (*P* > 0.05, Table 2). Furthermore, the independent t-test results indicated no significant difference between the mean scores of the HPM constructs in the intervention and control groups prior to the intervention (*P* > 0.05); however, three months after the intervention, a significant difference was observed between the mean scores of perceived benefits, perceived barriers, positive affect cues to behavior, negative affect cues to behavior, situational influences, and commitment to the action plan in the intervention and control groups (Table 3).

**Table 2:** Comparing control and intervention groups in terms of demographic characteristics

**Demographic characteristics**	**Intervention group**		**Control group**		***P*** ** value**
	**Number**	**Percent**	**Number**	**Percent**	
Age					0.666
Under 25 years	18	33.33	14	27.45	
26-35 years	32	59.25	31	60.78	
More than 36 years	4	7.4	6	11.76	
Education level					0.716
Under diploma	8	14.81	10	19.6	
Diploma	20	37.03	20	39.21	
Academic	26	14.48	21	41.17	
Number of children					0.569
No children	36	66.66	29	56.86	
One child	16	29.62	19	37.25	
Two or more children	2	3.07	3	5.8	
Insurance status					0.852
Yes	47	87.03	45	88.23	
No	7	12.96	6	11.76	
Occupation					0.283
Housewife	41	75.93	43	84.31	
Employed	13	27.04	8	15.68	

**Table 3:** Comparison of HPM constructs and behavior before and three months after the intervention in the intervention and control groups

	**Model components**	**Before intervention**		**After intervention**		***P*** ** value**	**Mean** **difference**
		**Mean **	**SD**	**Mean**	**SD**		
Perceived benefits	Control group	25.00	3.86	24.68	3.63	0.135	0.311
	Intervention group	25.07	4.50	26.57	3.67	0.001	-1.50
	*P* value	0.928		0.009			0.001
Perceived barriers	Control group	7.53	3.19	7.31	3.14	0.761	0.039
	Intervention group	6.59	3.84	5.81	3.59	0.001	0.777
	*P* value	0.297		0.025			0.001
Perceived self-efficacy	Control group	12.66	3.64	12.43	3.85	0.257	0.235
	Intervention group	12.62	4.23	13.55	3.98	0.004	-0.925
	*P* value	0.962		0.145			0.917
Positive affect cues to behavior	Control group	10.64	1.48	10.50	1.66	0.164	0.137
	Intervention group	11.11	1.34	11.29	1.34	0.159	-0.180
	*P* value	0.095		0.009			0.051
Negative affect cues to behavior	Control group	1.34	0.188	1.59	0.223	0.108	0.294
	Intervention group	1.79	1.61	1.40	1.51	0.026	0.026
	*P* value	0.147		0.006			0.029
Interpersonal influences(modeling)	Control group	11.35	3.46	11.01	2.92	0.920	0.019
	Intervention group	11.33	5.27	11.09	5.09	0.049	0.333
	*P* value	0.717		0.867			0.202
Interpersonal influences( social norms)	Control group	11.13	3.61	11.11	3.10	0.920	0.019
	Intervention group	11.35	3.46	11.01	2.92	0.192	0.333
	*P* value	0.075		0.665			0.702
Situational influences	Control group	17.54	4.55	17.27	4.40	0.159	0.274
	Intervention group	17.85	5.02	18.92	4.49	0.005	-1.07
	*P* value	0.747		0.060			0.002
Commitment to plan of action	Control group	4.15	1.88	4.05	1.92	0.280	0.098
	Intervention group	4.44	1.52	4.77	1.50	0.011	-0.333
	*P* value	0.390		0.034			0.007
Immediate competing demands and preferences	Control group	5.17	2.61	5.19	2.61	0.875	-0.019
	Intervention group	4.88	2.65	4.68	2.67	0.305	0.203
	*P* value	0.577		0.325			0.345

Table 4 presents the frequency and duration of tooth brushing in the intervention and control groups before and after the intervention. Brushing time for 2-3 minutes in the intervention group increased from 51.85% to 75.92% after the intervention. Before the intervention, there was no significant difference between the intervention and control groups regarding the duration of brushing (*P* = 0.561), while three months after the intervention, this difference was significant (*P* = 0.002) in this respect. However, no significant difference was found between the intervention and control groups regarding the tooth brushing frequency (*P* = 0.724) after the intervention (Table 4).

**Table 4:** Behavior in the intervention and control groups before and after the intervention

**Behavior/Category**	**Control**		**Intervention**		***P*** ** value**
	**Number**	**Percent**	**Number**	**Percent**	
Tooth brushing frequency/before					0.502
Never	0	0.00	0	0.00	
Once every two or three days	9	17.64	11	20.37	
Once a day	31	60/78	36	66.66	
Twice a day or more	11	21.56	7	12.96	
Tooth brushing frequency/after					0.724
Never	0	0.00	1	1.85	
Once every two or three day	7	13.72	7	12.96	
Once a day	34	66.66	33	61.11	
Twice a day or more	10	19.60	13	24.07	
Duration of tooth brushing/before					0.561
No brushing	0	0.00	0	0.00	
Less than 1 min	5	9.80	3	5.55	
1 min	24	47.05	23	42.59	
2-3 min and more	22	43.13	28	51.85	
Duration of tooth brushing/after					0.002
No brushing	0	0.00	0	0.00	
Less than 1 min	8	15.68	1	1.85	
1 min	20	39.21	12	22.22	
2-3 min and more	23	45.09	41	75.92	

## Discussion

 This study sought to evaluate the impact of an educational program according to the HPM on brushing behavior in pregnant women. HPM-based training enhanced the duration of tooth brushing behavior in the intervention group compared to the control group in the present study. However, there was no change in the frequency of tooth brushing. The results of some studies revealed improvements in preventive oral hygiene behaviors,^18,20,33^ whereas some studies found no considerable change in behavior. For instance, in the study conducted by Chawla et al,^34^ following oral health education in the local language via a PowerPoint presentation and dental referral, the participants’ knowledge and attitude improved considerably; however, their behavior (duration and frequency of brushing) represented no alteration. In the study by Adams et al,^35^ the intervention group had skill-based education twice for 15 minutes in two sessions, but the behavior (brushing and flossing) demonstrated no change. In general, it is highly challenging to modify habitual behaviors, including brushing teeth, which are no longer performed at the cognitive level but rather automatically. On the other hand, a shift in environmental conditions can act as a trigger, bringing a habitual behavior from a state of low control to one of high cognition and consciousness. In this case, the individual will consciously pay attention to the action and the nuances of completing it, resulting in the new health behavior becoming a habit.^36,37^ Thus, it appears that alterations in the desired behavior require more methodical and long-term planning.

 The findings of this study indicated that three months after the intervention, the perceived benefits of tooth brushing behavior were enhanced in the intervention group in comparison with the control group. These findings are in agreement with those of the study by Anderson et al,^38^ Shamsi et al,^23^ Rahmani et al,^24^ and Khani Jeihooni et al.^9^ Perceived benefits are perceptions of the positive outcomes of conducting a healthy behavior. In this case, individuals are likely to spend their resources and time on activities in which the possibility of positive outcomes is higher.^39^ The expectant mother’s understanding of the advantages of brushing her teeth might serve as an incentive to perform these behaviors regularly, increasing the likelihood of engagement in such activities. This improvement in the present study can be related to the education that was offered throughout the intervention regarding the advantages of brushing teeth with a favorable frequency and duration.

 Moreover, the results of this study demonstrated that three months after the intervention, the perceived barriers associated with tooth brushing behavior were considerably lower in the intervention group than in the control group. These findings are in line with those of similar studies.^19,22,23^ Barriers frequently provide incentives to avoid certain behavior.^32^ A review of the literature revealed that the barriers to engaging in hygienic behavior (tooth brushing) are nausea, misconceptions about brushing teeth, impatience and exhaustion throughout pregnancy, and a lack of family cooperation in performing household chores, and the solutions to overcome the barriers were presented in this study. In the revised HPM, perceived barriers to behavior have an impact on health-promoting behavior both directly and indirectly through lowering commitment to an action plan.^32^

 An additional finding of this study was that positive affect cues to behavior were promoted, while negative affect cues to behavior were reduced in the intervention group compared to the control group. These results are in agreement with those of the study by Dehdari et al^40^ on nutrient consumption in students’ breakfast and the results of the study by Goodarzi-Khoigani et al^41^ on the nutrition of expectant mothers based on HPM. Given that individuals are more inclined to repeat actions associated with positive feelings and to avoid behaviors related to negative feelings, it appears vital to address emotions associated with the behavior when developing interventions. Furthermore, the findings of this study confirmed that the intervention group was more committed to the action plan than the control group, which is in line with the findings of previous studies.^40,41^

 Some of the strengths of this study were the utilization of virtual education and the appropriate time and location for each individual. However, one of the study’s limitations was the assessment of behavior via self-report. Another constraint was that the pregnant woman’s attention was preoccupied with various matters linked to the health of the mother and the fetus throughout pregnancy, which might have affected the degree of involvement in group discussions about oral health. To modify this problem, telephone calls were used to increase mothers’ motivation to participate in group activities.

**Highlights**

- Brushing time for 2-3 minutes in the intervention group increased from 51.85% to 75.92% after the intervention.
- The tooth brushing frequency did not change after the educational intervention.
- Education based on the HPM may be effective in promoting the oral health behavior of pregnant women.

## Conclusion

 According to the findings of the present study, the perceived benefits and positive feelings toward the behavior were improved, leading to a reduction in the perceived barriers and the negative feelings towards the behavior, ultimately an increase in the duration of tooth brushing. Although it seems that more methodical and long-term planning is needed to increase the frequency of brushing (2 times a day). Eventually, more studies are suggested regarding the effect of educational programs with a theoretical framework on preventive oral health behaviors during pregnancy.

## Acknowledgements

 We thank Hamadan University of Medical Sciences for their support.

## Authors’ Contribution

**Conceptualization: **Saeid Bashirian.

**Data curation: **Maryam Barati.

**Formal analysis: **Salman Khazaei.

**Funding acquisition: **Saeid Bashirian.

**Investigation:** Maryam Barati.

**Methodology:** Majid Barati.

**Project administration: **Maryam Barati.

**Resources: **Samane Shirahmadi.

**Software: **Salman Khazaei.

**Supervision: **Leila Gholami.

**Validation: **Ensiyeh Jenabi.

**Visualization: **Majid Barati.

**Writing–original draft: **Maryam Barati.

**Writing–review & editing: **Maryam Barati.

## Competing Interests

 The authors declare no conflict of interests, financial or otherwise.

## Funding

 This project has been supported by the Research and Technology Deputy of Hamadan University of Medical Sciences (Grant Number: 9904242558).
